# Hearing Assessment in HIV-Exposed-Uninfected Infants

**DOI:** 10.3390/tropicalmed11050115

**Published:** 2026-04-27

**Authors:** Amanda Zanatta Berticcelli, Andréa Lúcia Corso, Pâmela Panassol, Leticia Petersen Schmidt Rosito, Roberta Rahal de Albuquerque, Letícia de Paula e Souza, Milena Lessa da Silva, Sady Selaimen da Costa, Luciana Friedrich

**Affiliations:** 1 Post-Graduate Program in Child and Adolescent Health, Federal University of Rio Grande do Sul, Porto Alegre 90035-903, Brazil; amanda.azb@gmail.com (A.Z.B.); alcorso@hcpa.edu.br (A.L.C.); pamela.panassol@gmail.com (P.P.); lsrosito@hcpa.edu.br (L.P.S.R.); sscosta@hcpa.edu.br (S.S.d.C.); 2 Department of Pediatrics, Federal University of Rio Grande do Sul, Porto Alegre 90035-903, Brazil; 3 Neonatology Service, Hospital de Clínicas de Porto Alegre, Porto Alegre 90035-903, Brazil; 4 Department of Otorhinolaryngology, Federal University of Rio Grande do Sul, Porto Alegre 90035-903, Brazil; 5 Otorhinolaryngology Service, Hospital de Clínicas de Porto Alegre, Porto Alegre 90035-903, Brazil; 6 Medical School, Federal University of Rio Grande do Sul, Porto Alegre 90035-903, Brazil; rralbuquerque@hcpa.edu.br (R.R.d.A.); leticiapsouza@hcpa.edu.br (L.d.P.e.S.); milenalsilva@hcpa.edu.br (M.L.d.S.)

**Keywords:** HIV, hearing loss, infant, auditory evoked potentials, vertical transmission, exposed uninfected

## Abstract

Background: Among the complications caused directly or indirectly by the Human Immunodeficiency Virus (HIV) are alterations in the auditory system. Children who are HIV-exposed but uninfected (HEU) appear to have a higher risk of hearing loss (HL) compared to their unexposed peers, but a lower risk than those infected with HIV. However, the literature remains inconclusive regarding this association. This study aims to evaluate the hearing function of HEU infants during the first months of life and to correlate these findings with maternal, gestational, and neonatal variables. Methods: This prospective cohort study included all HIV-exposed infants born in a quaternary hospital in southern Brazil between 2021 and 2023. Maternal, gestational, and neonatal data were collected, as well as the results of neonatal auditory screening. At approximately 6 months of age, otolaryngological and audiological assessments were performed, including wideband tympanometry and electrophysiological evaluation using Auditory Brainstem Response with frequency-specific stimuli. The prevalence of hearing loss refers to the number of infants affected. Results: Thirty-eight infants, with a mean age of 8 months (±3.3), completed the study. Of these, 1 (2.6%) presented with bilateral sensorineural HL, and 13 (34.2%) presented with conductive HL, with 6 cases being unilateral and 7 bilateral. No associations were found between hearing loss and maternal, gestational, or neonatal variables, except for maternal CD4 count, where higher CD4 cell counts were associated with an increased risk of conductive HL. Conclusion: The findings provide relevant data on auditory alterations in HEU infants, demonstrating a high prevalence of conductive HL. These results highlight the importance of monitoring the hearing of these children during the first years of life.

## 1. Introduction

Human Immunodeficiency Virus (HIV) represents a serious global public health issue [1]. Once inside the body, HIV affects the immune system, making the individual susceptible to opportunistic diseases. Due to the progressive impairment of the immune system, HIV infection constitutes a chronic health condition with social and economic impacts [2]. Over the past few decades, effective intervention strategies, such as global access to antiretroviral therapy (ART), safe childbirth and breastfeeding practices, and post-exposure prophylaxis administered to newborn (NBs), have successfully reduced HIV transmission from mother to fetus and newborn [3]. As a result, there has been a significant reduction in the rates of children infected through vertical transmission (VT), despite intrauterine exposure to the virus [1,4]. In Brazil, the HIV detection rate among pregnant women in 2022 was 3.1 per 1000 live births (LBs), with the Southern region accounting for 28.7% of all cases in the country. Rio Grande do Sul has the second highest detection rate of HIV in pregnancy (12% of all cases nationwide), with a rate of 7.9 cases per 1000 LBs. The city of Porto Alegre, the capital of Rio Grande do Sul, exhibits the highest detection rate among Brazilian capitals, at 17 per 1000 LBs, almost six times the national rate [5]. Children born to HIV-positive mothers but without HIV infection through VT are considered HIV-exposed but uninfected (HEU). According to data from the Joint United Nations Program on HIV/AIDS (UNAIDS), the cumulative global population of HEU in 2022 was estimated at approximately 16 million [6].

For a long time, researchers have sought to understand the impacts of HIV on the lives of infected children. Currently, with the growing population of HEU, efforts have focused on understanding how intrauterine exposure to the virus, maternal antiretroviral therapy (ART), and an immunologically adverse intrauterine environment may affect these children during the first months and years of life [4,7]. Recent studies suggest that intrauterine exposure to HIV can have negative repercussions throughout development into adulthood. Compared to unexposed children, HEU have a higher risk of morbidity and mortality, as well as impairments in growth and neurodevelopment, affecting motor, language, cognition, and behavior areas [8,9,10,11,12,13].

Among the numerous complications caused, either directly or indirectly, by HIV, alterations in the auditory system stand out. According to several studies, there appears to be an association between auditory abnormalities and HIV infection in the pediatric population, with hearing loss (HL) rates ranging from 24 to 39%, primarily conductive HL [9,14]. Most of these patients present with middle ear pathologies, such as otalgia, tympanic membrane perforations and otorrhea, with otitis media being the most common finding [15]. However, regarding HL in HEU children, the literature remains controversial. These children appear to have a higher risk of HL compared to their unexposed peers, but a lower risk than those infected with HIV [16]. It remains unclear how exposure to HIV and ART may impact the auditory system of these children [17].

Early diagnosis of HL in childhood is highly desirable, as its occurrence during the first years of life may lead to significant limitations in speech and language acquisition. Long-lasting conductive HL may have negative impacts on child development [18]. Moreover, without appropriate auditory rehabilitation, HL may result in adverse effects on psychomotor, cognitive, and educational development [9,19].

Despite this, there is no consensus regarding the need to monitor these children. The Joint Committee on Infant Hearing (JCIH) does not list HIV as an Indicator of Risk for Hearing Loss (IRHL) [20]. In Brazil, however, the Multiprofessional Committee on Auditory Health (COMUSA) includes HIV exposure in the list of RIHL and recommends monitoring auditory function until the third year of life [21]. Nevertheless, this regular follow-up is not implemented in the vast majority of health services in Brazil, despite public health policies on auditory health and existing national recommendations, thereby justifying initiatives that contribute to a better understanding of the audiological findings in this population. This study aims to evaluate the hearing function of HEU infants during the first months of life and to correlate these findings with maternal, gestational, and neonatal variables.

## 2. Materials and Methods

This was a prospective study, conducted at a public, university-based quaternary hospital in southern Brazil, which is a reference center for prenatal and neonatal care for HIV-positive pregnancies and HIV-exposed newborns (NBs). The sample was recruited based on convenience, including all live births (LBs) from HIV-positive mothers at the institution between May 2021 and March 2023, who continued follow-up after discharge at the institution’s Pediatric Infectious Disease Outpatient Clinic during the first year of life. Management of these newborns and their follow-up in the outpatient clinic were conducted according to the institution’s protocol.

Newborns with coexisting congenital infections (rubella, toxoplasmosis, cytomegalovirus [CMV], and syphilis), major congenital anomalies, or genetic syndromes that could result in hearing impairment were excluded. Preterm NBs with a gestational age (GA) of less than 34 weeks, patients who presented with perinatal asphyxia, hyperbilirubinemia at levels requiring exchange transfusion, and those who received ototoxic medications during the neonatal period (furosemide, vancomycin, or aminoglycosides) or required admission to the Intensive Care Unit for 5 days or longer were also excluded. Our institution has a quaternary Neonatal Intensive Care Unit with a genetic service that is a reference for the entire country, providing staff, imaging and laboratory exams capable of diagnosing and managing most genetic syndromes and congenital anomalies.

After birth, eligible NBs were identified, and their parents were invited to participate in the study. A urine sample from each newborn was collected for polymerase chain reaction (PCR) testing for cytomegalovirus (CMV) infection to avoid misattributing any HL due to CMV to HIV.

Maternal and gestational data related to HIV infection and treatment were collected through electronic medical record review. Data related to pregnancy, delivery and perinatal variables were also collected, along with information on neonatal hearing screening (NHS). According to the institution’s protocol, HIV-exposed NBs underwent a transient evoked otoacoustic emissions (TEOAE) before hospital discharge. As these newborns had no other indicators of risk for IRHL, auditory brainstem response (ABR) testing was only performed in cases where TEOAE failed.

All NBs received ART prophylaxis for 28 days, in accordance with the guidelines of the Brazilian Ministry of Health (MoH) [7,22]. After discharge, they were referred to the Pediatric Infectious Disease Outpatient Clinic of the same hospital for routine clinical and laboratory follow-up.

The risk of VT was considered “high” or “low” according to the Ministry of Health (MoH) protocol [7,22]. NBs were classified as low risk for VT when their mothers used ART regularly, with good adherence, had initiated treatment during the first half of pregnancy, and had an undetected viral load (VL) in the third trimester. All other pregnancy scenarios were classified as high risk for VT. ART prophylaxis for the NBs was given according to the VT risk [7,22].

Newborns were also classified as appropriate for gestational age (AGA), small for gestational age (SGA), or large for gestational age (LGA), according to the INTERGROWTH-21st standards [23]. According to the routine outpatient follow-up, the infants entered the second phase of the study, which was conducted at a private clinic, where they were referred for the following procedures. The audiologic tests were blind, conducted without knowledge of maternal and perinatal data:

- Otolaryngological evaluation, including a focused history related to the otolaryngological physical examination;
- Audiological evaluation, including wideband tympanometry and auditory brainstem response (ABR) using frequency-specific stimuli (FS-ABR);
- Wideband tympanometry was performed using Titan equipment from Interacoustics. Tympanometric curves with probe tones of 1000 Hz and 226 Hz were analyzed according to age-specific recommendations. For the 1000 Hz probe, curves were classified as normal or abnormal [24]. When using the 226 Hz probe, based on the values found for compliance and tympanometric gradient, the responses were classified as type A, B, or C curves [25]. Additionally, acoustic reflexes were assessed and classified as “present” or “absent” based on the automatic analysis of the equipment.

Frequency-specific auditory brainstem response (FS-ABR) was conducted using the Eclipse EP25 equipment (Interacoustics) and included assessment of auditory pathway integrity and electrophysiological threshold estimation. Tests were conducted during natural sleep, with the infant positioned on the lap of the mother or caregiver. Electrodes were placed on the forehead (Fz [frontal—zero degrees—midline] and Fpz [pre-frontal—zero degrees—midline]) and on the right (A2) and left (A1) mastoids, according to the institution’s protocols.

The integrity of the auditory pathway was assessed using a click stimulus in alternating polarity and filters set from 100 to 3000 Hz. A total of 2000 stimuli were presented at a rate of 27.1 per second, at an intensity of 80 dB nHL in monaural mode through insert earphones. The absolute latencies of waves I, III, and V were analyzed, as well as the interpeak latencies I-III, III-V, and I-V. The electrophysiological threshold estimation was performed with the NB CE-Chirp LS^®^ stimulus and alternating polarity at frequencies of 500, 1000, 2000, and 4000 Hz via air conduction. The electrophysiological threshold was considered the lowest intensity at which wave V was present, observed in at least two recordings to ensure replicability. Thresholds of 35, 30, 30, and 25 dB HL, respectively, were considered normal.

In cases of abnormal electrophysiological thresholds via air conduction, bone conduction testing was performed using the B71 bone transducer at frequencies of 2000 and 500 Hz in an ascending manner, starting at 30 and 20 dB nHL, respectively. The electrophysiological threshold was considered the lowest value at which wave V was present.

After the evaluations were concluded, findings were classified as normal hearing (when tympanometry and air and bone conduction were normal), conductive abnormality (when tympanometry and air conduction were altered, but bone conduction was normal) or sensorineural abnormality (when there was normal tympanometry but altered air and bone conduction).

All 38 infants were classified as HIV-uninfected after three undetectable VL tests during the first year of life, along with a nonreactive chemiluminescence serological test at 12–18 months of age [7].

Data were entered into an Excel spreadsheet and subsequently analyzed using the Statistical Package for the Social Science (SPSS) version 29.0. Quantitative variables with a symmetric distribution were described using mean and standard deviation, whereas those with an asymmetric distribution were described using the median and interquartile range. Categorical variables were analyzed using absolute and relative frequencies. To evaluate the association between HL and maternal, gestational, and neonatal variables, a bivariate analysis was conducted. A significance level of 5% (*p* < 0.05) was adopted. The project was approved by the institution’s Research Ethics Committee (Number 2021-0130), and all mothers or legal guardians provided written informed consent.

## 3. Results

### 3.1. Sample Characterization

**Figure 1:**
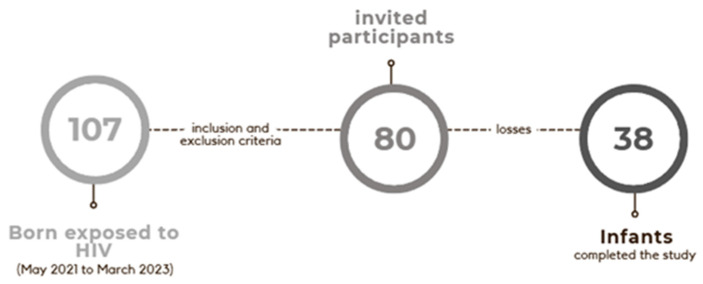
Flowchart demonstrating the sample of the study.

Between 1 May 2021 and 31 March 2023, a total of 5931 LBs occurred at the study hospital. Of these, 107 births were HIV-exposed, resulting in an incidence of HIV-exposed NBs of 1.8 per 1000 LBs. Among the 107 HIV-exposed NBs, 27 met the exclusion criteria, and 80 were eligible for the study and invited to participate. Of these 80 patients, 38 (47.5%) completed all planned stages of the study. All NBs initiated ART prophylaxis within the first hours of life and were not breastfed. Figure 1 presents a flowchart demonstrating the sample of the study. The complete characterization of the sample is presented in Table 1.

### 3.2. Neonatal Hearing Screening

All patients underwent Neonatal Hearing Screening (NHS) before hospital discharge. Of the 38 patients, 31 passed the initial screening with TEOAE and 7 were referred for retesting; 4 passed the retesting and 3 failed and were referred for automated auditory brainstem response (AABR). All 3 patients failed the AABR and were subsequently referred for comprehensive audiological evaluation in the outpatient otorhinolaryngology clinic in the same hospital.

### 3.3. Hearing Testing

Infants underwent the second phase of the study at a mean age of 7 ± 3.3 months. Tympanometric findings and ABR results were analyzed and categorized into evaluation of integrity (absolute and interpeak latencies) and electrophysiological thresholds. Table 2 presents the values of absolute latencies and interpeak latencies along with the tympanometric findings.

The absolute values of waves I, III, and V and interpeak intervals were analyzed according to age-specific normative values defined by the equipment and classified as “normal” or “delayed latency”. In 23 infants (60.5%), the values were considered normal. Block delay was observed in 13 infants (34.2%), and the delay of waves III and V and/or interpeak latencies I–III and I–V was noted in 2 infants (5.3%). Electrophysiological thresholds were within normal limits at all frequencies assessed in 24 infants (63.1%). In 14 infants (36.8%), there was an abnormality in the electrophysiological threshold at least at one frequency, with 13 having deviant air conduction (AC) and normal bone conduction (BC), and 1 patient having deviant values for both AC and BC.

**Figure 2:**
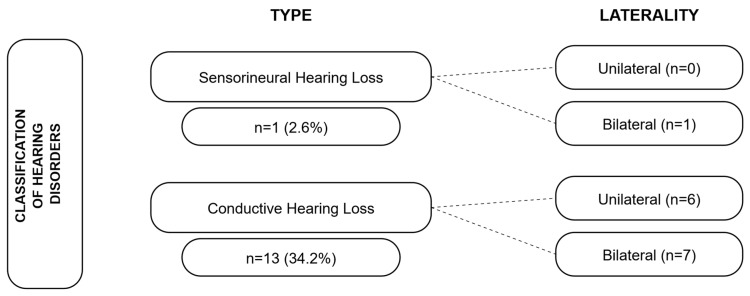
Classification of alterations according to auditory evaluation.

Among the 24 infants with normal electrophysiological thresholds, 2 (5.3%) showed delayed latencies of waves III and V and/or inter-peak latencies I–III and I–V. For the 14 infants with abnormal thresholds, the findings were classified according to the type of alteration as follows: “conductive HL” (when tympanometry and AC were abnormal, with normal BC) and “sensorineural HL” (when tympanometry was normal, but both AC and BC were abnormal). These results are shown in Figure 2.

All patients who attended the follow-up were evaluated by an otolaryngologist. All infants with normal audiological examinations, as well as the only infant with sensorineural HL, had normal otoscopy findings. Infants with conductive HL presented with otoscopic abnormalities of the middle ear, such as otitis media with effusion. There were no discordant test results regarding tympanometry and ABR.

### 3.4. Association Between Hearing Loss and Maternal Variables

Table 3 presents the analysis of the association between HL and maternal factors (timing of maternal HIV diagnosis), gestational variables (regular ART use, VL quantification, CD4 count, and mode of delivery), and neonatal factors (weight adequacy for GA and VT risk). For analysis purposes, the only infant who presented with sensorineural HL was excluded, as it was not possible to test for CMV infection in this patient, making it impossible to determine whether HL was caused by HIV exposure or CMV infection. One pregnant woman was unaware of her HIV diagnosis. Therefore, the association analyses included 37 infants, of whom 24 had normal examinations and 13 had conductive HL, except for the “HIV diagnosis” variable, which included 36 infants.

A significant association was found between HL and maternal CD4 cell count, with higher maternal CD4 cell counts being associated with an increased risk of conductive HL. No other associations were statistically significant. However, maternal viral load and ART use during pregnancy had borderline significance.

### 3.5. Status of HIV Infection

All evaluated infants were confirmed as HIV-uninfected through three undetectable VL tests during the first year of life, as well as a negative anti-HIV serological test performed after 12 months of age, in accordance with MoH guidelines for excluding infection in HIV-exposed infants.

### 3.6. CMV Data

Of the 38 infants who completed the study, 13 (34.2%) provided a urine sample for CMV PCR testing. All the results were negative for CMV. The other 25 infants who did not undergo CMV testing were discharged early from the hospital, without sufficient time to collect the urine sample.

### 3.7. Post Hoc Analysis

A post hoc analysis was conducted to determine the power of the study after the losses to follow-up. Considering our 25 patients with maternal CD4 above 500 copies/mL and 8 patients with maternal CD4 below 500 copies/mL, this study has a calculated power of 84.1% to detect a difference of 52% in the prevalence of hearing loss, with α = 0.05.

## 4. Discussion

This study found conductive HL in 34.2% of HIV HEU infants, as well as 1 infant (2.6%) with bilateral sensorineural HL, despite the favorable prognostic characteristics of the sample (full-term births, appropriate weight for GA, high Apgar scores). Most pregnant women were diagnosed with HIV before pregnancy, received regular antenatal care, used ART regularly, had an undetectable VL in the third trimester, and had adequate CD4 cell counts.

Studies in the literature describe middle ear pathologies in children infected with HIV, primarily conductive abnormalities, in up to 18–25% of patients [7,14,15,18]. However, regarding HEU NBs and infants, the literature remains scarce and limited to the first few months of life, making it difficult to draw conclusions about auditory development [26]. To date, there is no consensus regarding the association between HEU infants and HL.

Some authors have reported normal NHS in this population, suggesting normal cochlear function at birth. However, the absence of cochlear alterations observed in NHS does not exclude the possibility of later auditory impairment [27]. This study found altered NHS in 3 NBs (7.9%), considering both test and retest. Of these, 2 (5.3%) subsequently showed normal diagnostic hearing tests (tympanometry and ABR) and 1 (2.6%) was diagnosed with moderate bilateral sensorineural HL and was referred for auditory rehabilitation. This patient was subsequently excluded from the association analyses, as no CMV detection test was performed, making it impossible to determine whether the cause of HL was associated with HIV exposure.

A previous study evaluating auditory responses in HEU using ABR did not find a significant difference in the prevalence of HL between HEU and non-exposed NBs [26]. The same authors reported higher mean auditory thresholds at 1, 3, 6, and 9 months of age in HEU infants compared to non-exposed ones, using click stimuli in ABR [28].

Our study is among the first to evaluate HL in HEU infants during the first months of life using FS-ABR. We found a 36.8% prevalence of hearing impairment, predominantly conductive HL (13 of 14 cases—92.8%). All the affected infants had abnormal otoscopy, mainly with middle ear effusion, even without acute otitis media. These findings suggest that, even in the absence of HIV infection, exposure to the virus may interfere in some way with hearing function. It can be hypothesized that HIV exposure can affect the immune system of these children, making them more susceptible to middle ear infections in the first months of life compared to the non-exposed population. The causes of this potential immunological alteration are not yet fully understood. One hypothesis is placental inflammation, which could result in reduced transplacental antibody transfer to the fetus, thereby impairing immune function. Some studies have reported differences in CD4 maturation, reduced thymus size, and frequent neutropenia in these patients [7]. This altered immune response also appears to have a direct association with maternal VL at the time of delivery, with children born to mothers with a VL greater than 1000 copies/mL being exposed to a higher risk of immunosuppression due to compromised development of their immune system [7]. Additionally, the absence of breastfeeding in these patients could be an additional cause of immune deficiency, although the exact impact of this situation has not yet been fully clarified [7].

Maternal, gestational, and neonatal factors were analyzed to identify potential associations with the HL detected in the study. No association was found between HL and the timing of HIV diagnosis or the mode of delivery. There was also no association between HL and ART use, which is consistent with previous studies [29,30]. Little is still known about the effects of ART on the auditory health of children and adolescents. However, the association of maternal VL and ART use with hearing loss had borderline significance in the statistical analysis, possibly due to the small sample size.

The present study found no significant association between maternal VL quantification and auditory alterations. It is worth noting that most pregnant women included in the study had an undetectable VL (71.1% of cases), and 68.4% of them had a CD4 count above 500 cells/mm^3^. These data are consistent with appropriate prenatal care, in accordance with the guidelines of the Brazilian MoH [7]. A positive association was found between conductive HL and a higher maternal CD4 count, a finding not reported in the reviewed literature. For example, studies by Fasunla et al. [26,28], which assessed HEU NBs, found no significant relationship between HL and maternal CD4 count.

The main limitation of this study was the number of patients who were able to complete the second phase of the research. The number of losses reflects the difficulty in following this population, whether due to socioeconomic and mobility challenges, parents’ lack of understanding that HEU infants may still have clinical and laboratory abnormalities resulting from an unfavorable immunological condition, or even due to the stigma that HIV exposure causes in families. Additionally, this study was conducted during the COVID-19 pandemic, which further hindered patients’ access to the healthcare system.

Another limitation of the study was the absence of a control group of non-HIV-exposed infants, which would have allowed comparison with the HEU group, as middle ear alterations occur in the general pediatric population during the first years of life [9,31]. To reduce the occurrence of confounding biases, the sample in the present study consisted of infants with no other risk factors for HL besides intrauterine HIV exposure. We excluded preterm infants, significant congenital anomalies and infants with coinfections like congenital syphilis, CMV and neonatal herpes infection. It is important to note that most HIV-exposed infants born in our institution are born at term and healthy.

Additionally, it was not possible to perform PCR tests for CMV in all recruited patients, preventing the reliable exclusion of HL due to this infection. However, although only 34.2% of the sample was tested, all of these patients had negative results. The HL detected in the present study was conductive HL, which is not consistent with sensorineural HL caused by congenital CMV infection. The only infant with sensorineural HL was excluded from the subsequent analyses. This difficulty in collecting urine samples for CMV PCR testing was primarily due to the early discharge of most of the NBs in the study, who were clinically stable and remained only in the Rooming-In Unit, often being discharged before the research team could request and collect the test. Knowing that the global prevalence of congenital CMV is around 1% and is even higher in HIV-exposed newborns, and since we collected the PCR exam in 34% of our sample with no detected CMV, our hypothesis of hearing abnormalities due to HIV exposure is reliable. Not having detected CMV coinfection in most of the infants with abnormal hearing tests makes it easier to infer that these hearing abnormalities were probably due to HIV exposure.

## 5. Conclusions

This study demonstrated a high prevalence of conductive HL in HEU infants with no other IRHL. Larger and controlled studies are important to confirm these findings, and the importance of auditory monitoring for these children during the first years of life is evident.

## Figures and Tables

**Table 1:** Maternal and neonatal data of the sample (n = 38).

Variables	n = 38
**Maternal** age (years) *	31.1 ± 5.8
Gestational age (weeks) *	38.5 ± 1.9
**Antenatal appointments**	
<6 appointments	10 (26.3%)
≥6 appointments	27 (71.1%)
Unknown	1 (2.6%)
**Maternal HIV diagnosis**	
Before pregnancy	32 (84.2%)
During pregnancy	5 (13.2%)
Data unknown	1 (2.6%)
**Maternal ART use**	
Regular	30 (78.9%)
Irregular/Absent	8 (21.1%)
**Maternal VL in the 3rd trimester**	
>1000 copies/mL	2 (5.3%)
500–1000 copies/mL	0
40–500 copies/mL	4 (10.5%)
<40 copies/mL	5 (13.2%)
Undetectable	27 (71.1%)
**CD4 count during pregnancy**	
>500 cells/mm3	26 (68.4%)
<500 cells/mm3	8 (21.1%)
Unknown	4 (10.5%)
**Intrapartum intravenous AZT**	
Yes	35 (92.1%)
No	3 (7.9%)
**Mode of delivery**	
Vaginal	17 (44.7%)
Cesarean	21 (55.3%)
Birth weight (grams) *	**3006** ± 585.6
**Newborn gender**	
Male	23 (60.5%)
** Female**	**15 (39.5%)**
**Weight/Gestational age classification**	
AGA	26 (68.4%)
SGA	10 (26.3%)
LGA	2 (5.3%)
**Risk of HIV vertical transmission**	
Low risk	26 (68.4%)
High risk	12 (31.6%)
**Urinary CMV PCR in the newborn**	
** Detected**	**0**
** Not detected**	**13 (34.2%)**
** Not performed**	**25 (65.8%)**

Legend: * mean ± standard deviation; HIV: Human Immunodeficiency Virus; ART: antiretroviral therapy; AZT: Zidovudine; AGA: appropriate for gestational age; SGA: small for gestational age; LGA: large for gestational age; CMV: Cytomegalovirus; PCR: polymerase chain reaction.

**Table 2:** Findings of tympanometric curves and values of absolute latencies of waves I, III, and V and interpeak latencies I–III, I–V, and III–V (n = 38).

	Right Ear							Left Ear						
nº	Tymp	I	III	V	I–III	I–V	III–V	Tymp	I	III	V	I–III	I–V	III–V
1	A	1.40	4.27	6.33	2.87	4.93	2.06	A	1.33	3.93	6.16	2.60	4.80	2.20
2	A	1.40	4.13	6.27	2.73	4.87	2.14	A	1.47	4.20	6.13	2.73	4.66	1.93
3	C	1.67	4.00	6.07	2.33	4.40	2.07	C	1.53	4.13	6.20	2.60	4.67	2.07
4	A	1.33	4.13	6.00	2.80	4.67	1.87	A	1.27	4.00	6.13	2.73	4.86	2.13
5	A	1.40	4.00	5.93	2.60	4.53	1.93	A	1.40	4.00	5.87	2.60	4.47	1.87
6	A	1.33	4.07	6.40	2.74	5.07	2.33	A	1.27	4.00	6.27	2.73	5.00	2.27
7	A	1.27	3.87	5.80	2.60	4.53	1.93	C	1.20	3.80	5.87	2.60	4.67	2.07
8	A	1.67	4.13	6.13	2.53	4.53	2.00	A	1.53	4.07	5.93	2.54	4.40	1.86
9	A	1.40	4.07	6.07	2.67	4.67	2.00	A	1.47	3.93	6.00	2.46	4.53	2.07
10	A	1.47	3.87	5.87	2.40	4.40	2.00	A	1.47	3.93	5.93	2.46	4.46	2.00
11	A	1.40	3.87	6.13	2.47	4.73	2.26	A	1.27	4.07	6.20	2.80	4.93	2.13
12	A	1.40	3.53	5.60	2.13	4.20	2.07	A	1.40	3.53	5.47	2.13	4.07	1.94
13	A	1.33	4.00	6.07	2.67	4.74	2.07	A	1.33	4.07	6.27	2.74	4.94	2.20
14	A	1.47	4.13	6.33	2.66	4.86	2.20	A	1.27	4.07	6.20	2.80	4.93	2.13
15	A	1.27	3.73	5.60	2.46	4.33	1.87	A	1.33	3.73	5.67	2.40	4.34	1.94
16	A	1.67	4.13	6.20	2.46	4.53	2.07	A	1.53	4.13	6.27	2.60	4.74	2.14
17	C	1.60	4.20	6.33	2.60	4.73	2.13	A	1.40	3.93	6.13	2.53	4.73	2.20
18	A	1.40	3.87	5.80	2.47	4.40	1.93	A	1.33	3.80	5.80	2.47	4.47	2.20
19	A	1.33	3.67	5.80	2.34	4.47	2.13	A	1.40	3.67	5.80	2.27	4.40	2.13
20	A	1.53	4.07	5.93	2.54	4.40	1.86	A	1.60	4.07	6.13	2.47	4.53	2.06
21	A	1.40	4.00	5.87	2.60	4.47	1.87	A	1.33	4.00	5.80	2.67	4.47	1.80
22	A	1.47	3.80	5.67	2.33	4.20	1.87	A	1.33	3.80	5.60	2.47	4.27	1.80
23	A	1.37	4.20	6.47	2.83	5.10	2.27	A	1.37	4.20	6.70	2.83	5.33	2.50
24 *	A	1.33	4.07	6.27	**2.74**	**4.94**	2.20	A	1.47	4.27	6.33	**2.80**	**4.83**	2.06
25 *	A	1.47	**4.40**	**6.80**	**2.93**	**5.33**	2.40	A	1.60	**4.40**	**6.80**	**2.80**	**5.20**	2.40
26 **	B	**2.00**	**4.47**	**6.80**	2.47	4.80	2.33	B	**2.80**	**5.53**	**7.33**	2.73	4.53	1.80
27 **	B	**2.47**	**4.80**	**6.73**	2.33	4.26	1.93	B	**2.80**	**4.93**	**6.73**	2.13	3.93	1.80
28 **	B	**1.93**	**4.47**	**6.73**	2.54	4.80	2.26	B	**1.93**	**4.47**	**6.80**	2.54	4.87	2.33
29 **	B	**2.27**	**4.47**	**6.73**	2.20	4.46	2.26	B	**1.93**	**4.53**	**6.73**	2.60	4.80	2.20
30 **	B	**2.13**	**4.60**	**6.40**	2.47	4.27	1.80	B	**2.20**	**4.60**	**6.40**	2.40	4.20	1.80
31 **	B	**1.87**	**4.40**	**6.40**	2.53	4.53	2.00	B	**1.87**	**4.47**	**6.47**	2.60	4.60	2.00
32 **	B	**2.20**	**4.47**	**6.40**	2.27	4.20	1.93	B	**2.47**	**4.80**	**6.67**	2.33	4.20	1.87
33 ***	B	**2.33**	**4.53**	**6.73**	2.20	4.40	2.20	A	1.47	4.20	6.00	2.73	4.53	1.80
34 ***	B	**2.33**	**4.73**	**7.07**	2.40	4.74	2.34	C	1.70	4.20	6.47	2.50	4.77	2.27
35 ***	B	**2.20**	**4.67**	**6.40**	2.47	4.20	1.73	C	1.47	3.93	5.93	2.46	4.46	2.20
36 ***	A	1.40	4.27	6.53	2.87	5.13	2.26	B	**2.00**	**4.80**	**6.93**	2.80	4.93	2.13
37 ***	A	1.60	4.20	6.00	2.60	4.40	1.80	B	**2.60**	**5.07**	**7.13**	2.47	4.53	2.06
38 ***	C	1.60	4.27	6.33	2.67	4.73	2.06	B	**1.93**	**4.47**	**6.40**	2.54	4.47	1.93

**Legends:** Tymp: Tympanometric curve. Abnormal values in bold and underlined. Values of absolute latencies and interpeak latencies are presented in milliseconds. * Delay of waves III and V and/or interpeaks and I–III and I–V. ** Block delay of latencies of waves I, III, and V in both ears. *** Block delay of latencies of waves I, III, and V in one ear.

**Table 3:** Association between hearing loss (HL) and maternal, gestational, and neonatal variables.

	Auditory Evaluation		
	Normal	Altered	*p* Value *
**HIV diagnosis (n = 36)**			
Before pregnancy	19	12	0.634
During pregnancy	4	1	
**ART use (n = 37)**			
Regular	19	10	0.796
Irregular/Absent	5	3	
**Maternal CD4 (n = 33)**			
>500 cells/mm3	12	13	**0.012**
<500 cells/mm3	8	0	
**Maternal viral load (n = 37)**			
>1000 copies/mL	2	0	0.565
500–1000 copies/mL	0	0	
40–500 copies/mL	3	1	
<40 copies/mL	2	3	
Undetectable	17	9	
**Mode of delivery (n = 37)**			
Vaginal	12	5	0.731
Cesarean	12	8	
**Weight for gestational age classification**			
AGA	19	7	0.266
SGA	4	5	
LGA	1	1	
**VT risk (n = 37)**			
Low	18	7	0.274
High	6	6	

**Legend:** * Fisher’s test; HIV: Human Immunodeficiency Virus; ART: antiretroviral therapy; AGA: appropriate for gestational age; SGA: small for gestational age; LGA: large for gestational age; VT: vertical transmission.

## Data Availability

The authors declare that all data generated or analyzed during this study are included in this published article. Some personal patients’ data are not publicly available but are available from the corresponding author on reasonable request.

## Abbreviations

The following abbreviations are used in this manuscript:

AABR	Automatic Auditory Brainstem Response
ABR	Auditory Brainstem Response
AC	Air conduction
AGA	Adequate for Gestational Age
ART	Antiretroviral Therapy
BC	Bone Conduction
CMV	Cytomegalovirus
COMUSA	Multiprofessional Committee on Auditory Health (Portuguese)
FS-ABR	Frequence-Specific Automatic Brainstem Response
GA	Gestational Age
HEU	HIV-Exposed Uninfected
HIV	Human Immunodeficiency Virus
HL	Hearing Loss
Hz	Hertz
IRAD	Indicator of Risk for Auditory Deficiency
JCIH	Joint Committee on Infant Hearing
LB	Live Birth
LGA	Large for Gestational Age
MoH	Ministry of Health
NB	Newborn
NHS	Neonatal Hearing Screening
PCR	Polymerase Chain Reaction
SGA	Small for Gestational Age
SPSS	Statistical Package for Social Sciences
TEOAE	Transient Evoked Otoacustic Emissions
UNAIDS	United Nations Programme on HIV/AIDS
VL	Viral Load
VT	Vertical Transmission
